# Connexins: substrates and regulators of autophagy

**DOI:** 10.1186/s12860-016-0093-9

**Published:** 2016-05-24

**Authors:** Jegan Iyyathurai, Jean-Paul Decuypere, Luc Leybaert, Catheleyne D’hondt, Geert Bultynck

**Affiliations:** KU Leuven, Laboratory of Molecular and Cellular Signaling, Department Cellular and Molecular Medicine, Campus Gasthuisberg O/N-I bus 802, Herestraat 49, B-3000 Leuven, Belgium; KU Leuven, Laboratory for Membrane Trafficking, Department of Human Genetics, and VIB-Center for the Biology of Disease, Campus Gasthuisberg, O/N-IV, 7.159, Herestraat 49, 3000 Leuven, Belgium; Ghent University, Physiology Group, Department of Basic Medical Sciences, 9000 Ghent, Belgium

**Keywords:** Autophagy, Connexins, Degradation, Regulation

## Abstract

Connexins mediate intercellular communication by assembling into hexameric channel complexes that act as hemichannels and gap junction channels. Most connexins are characterized by a very rapid turn-over in a variety of cell systems. The regulation of connexin turn-over by phosphorylation and ubiquitination events has been well documented. Moreover, different pathways have been implicated in connexin degradation, including proteasomal and lysosomal-based pathways. Only recently, autophagy emerged as an important connexin-degradation pathway for different connexin isoforms. As such, conditions well known to induce autophagy have an immediate impact on the connexin-expression levels. This is not only limited to experimental conditions but also several pathophysiological conditions associated with autophagy (dys)function affect connexin levels and their presence at the cell surface as gap junctions. Finally, connexins are not only substrates of autophagy but also emerge as regulators of the autophagy process. In particular, several connexin isoforms appear to recruit pre-autophagosomal autophagy-related proteins, including Atg16 and PI3K-complex components, to the plasma membrane, thereby limiting their availability and capacity for regulating autophagy.

## Background

### A short overview of the autophagy process

Autophagy (meaning “to eat oneself”) is a term to describe the intracellular processes responsible for the delivery of the cell’s own macromolecules to the lysosomes (or vacuoles) for degradation and recycling [1]. Three main types of autophagy can be distinguished based on their mechanism of delivery. In microautophagy, direct delivery happens through invagination of the lysosomal membrane, while chaperone-mediated autophagy relies on molecular chaperones and lysosome-associated membrane protein 2 LAMP2 to guide misfolded proteins to the lysosomes. The best studied type of autophagy, macroautophagy (hereafter referred to as “autophagy”), involves the transport and delivery of intracellular macromolecules or organellar fragments to the lysosomes inside double-membranous vesicles (autophagosomes). Due to their relatively large size (0.5 – 1.5 μm in mammalian cells [2]), autophagosomes were already discovered by transmission electron microscopy in the early sixties and the term “autophagy” launched by Nobel Prize-winning lysosomal expert Christian de Duve in 1963 [3]. However, its importance became only acknowledged in 1999, when Levine and co-workers discovered that a critical autophagy protein, Beclin 1, is a tumor suppressor [4]. This seminal work and further investigation established autophagy as a crucial player in cellular life and death during stress and disease. Indeed, upon nutritional, hypoxic, chemical or mechanical stress, autophagy is often stimulated, leading to enhanced degradation and recycling of molecular components. This way, possibly toxic damaged macromolecules, aggregates or organelles can be swiftly removed, especially those that are too large to be degraded by the proteasome or when the proteasomal degradation is blocked [5]. Moreover, lysosome-dependent autophagic recycling creates a new pool of cellular building blocks to be incorporated into the required anti-stress proteins. This is particularly convenient during nutrient deprivation, when non-selective bulk recycling of macromolecules ensures the availability of amino acids and cellular survival. In contrast, specific autophagic degradation of protein aggregates or organelles (e.g. mitochondria, a process termed mitophagy) is mediated by specific proteins tagging and sequestering polyubiquitin chains or damaged organelles. As such, it is clear that autophagy is a self-protective process that removes possible toxic cell death-inducing signals and enhances survival. However, in certain situations, autophagy may actually provoke cell death. Although autophagy-dependent cell death is likely less frequent than pro-survival autophagy and while its mechanistic details are still unclear, it is a clinically relevant phenomenon (e.g. upon ischemia) [6–8].

**Fig. 1:**
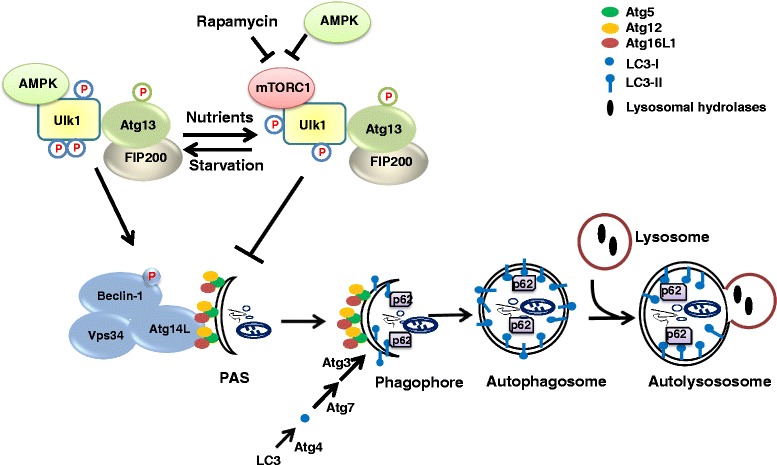
Macroautophagy is a major recycling pathway for long-lived proteins, protein aggregates and organelles. Autophagy induction is controlled by the AMPK/mTOR-signaling axis. In nutrient-rich conditions, AMPK activity is low and mTOR activity is high, thereby suppressing the autophagic pathway. Upon nutrient starvation and/or energy deprivation, AMPK activity is high, causing the inhibition of mTOR (directly and indirectly via the TSC1/2-Rheb GTPase pathway) and activation of the ULK1 complex involving a complex network of phosphorylations. mTOR inhibition and autophagy activation can also be achieved in nutrient-rich conditions using chemical mTOR inhibitors like rapamycin. Activation of the ULK1 complex contributes to the activation of the class III PI3K complex (Atg14L/Beclin 1 (Atg6)/Vps34 and the Vps34-regulatory protein Vps15 (not shown)) by phosphorylating Beclin 1, producing PtdIns(3)P from PtIns at the pre-autophagosomal structures (PAS) necessary for the formation of the phagophore. This structure then elongates into a double-membranous vesicle, the autophagosome, by recruitment of the Atg12/Atg5/Atg16 complex and the lipidation of LC3-I into phosphatidylethanolamine-conjugated LC3-II. LC3-II formation involves the production of LC3-I by Atg4-mediated proteolytic cleavage of LC3 and the action of E1-like ligases (Atg7), E2-like ligases (Atg3) and E3-like ligase complexes (Atg12/Atg5/Atg16L1). Furthermore, ubiquitin-binding proteins like p62 can bind LC3, thereby linking ubiquitinated proteins to LC3-II and targeting them for autophagic degradation. The Atg12/Atg5/Atg16 complex present in the outer membrane dissociates from mature autophagosomes, which then fuse with the lysosomes, degrading the inner membrane and the cargo present in the autophagosomes via luminal hydrolysases and subsequently releasing the digested material via permeases back into the cytosol. The fusion of autophagosomes with lysosomes can also involve endosomes, leading to intermediate amphisome formation

To ensure that the formation, transport and fusion of autophagosomes is correctly executed, a network of molecular pathways and proteins are involved (Fig. 1). Many of these players are shared with the endocytic pathway [9]. However, the autophagic pathway also contains a set of autophagy-specific proteins (Atg proteins) that function in the formation of the double membranous structure (phagophore), its subsequent elongation and closure into an autophagosome and the sequestration of its cargo [10, 11]. The origin of the phagophore is still debated, with different organelles postulated as candidates as source of this lipid bilayer [12]. This likely depends on the type of stress that induces autophagy and the material that needs to be degraded (specific organelles or structures or non-selective cytoplasmic in bulk degradation). At the endoplasmic reticulum (ER), clear observations have been made of the phagophore gradually originating and protruding from PtdIns(3)P-rich sites called omegasomes [13, 14]. This is mediated by the phosphoinositide 3-kinase (PI3K) Class III complex, consisting of (among others) Vps34, Vps15, Beclin 1 (Atg6) and Atg14L, which phosphorylates PtdIns to PtdIns(3)P. The PtdIns(3)P signal recruits several proteins (e.g. WIPI1 and2 (Atg18) [15] that induce the protrusion of the phagophore from the ER membranes. PI3K Class III activity is regulated by the nature of its interacting partners (including anti-apoptotic proteins Bcl-2 and Bcl-X_L_ [16, 17]) and by the ULK1/2 complex [18]. The latter consists of ULK1/2, Atg13 and FIP200 and mediates the response of autophagy towards nutrient availability and energy status, as it is regulated by the nutrient sensing kinase mTOR and the energy sensing AMP-dependent kinase AMPK, respectively. mTOR inhibits the ULK1/2 complex by phosphorylation when amino acids and growth factors are abundant. When nutrients are scarce however, mTOR activity is repressed and ULK1/2 active. Upon energy deprivation, AMPK phosphorylates ULK1/2 at different sites than mTOR, activating the complex [19], which stimulates PI3K Class III and triggers the formation of the phagophore. AMPK also inhibits mTOR activity by phosphorylating TSC1/TSC2 (tuberous sclerosis complex 1 and 2), thereby suppressing RHEB (Ras homologue enriched in brain) activity [20]. Further elongation of the phagophore is mediated by delivery of novel lipids transported by lipid carrier protein Atg9 [21], and by two ubiquitin-like reaction processes that are dependent on Atg7 [10, 22]. First, Atg5 is covalently linked Atg12 by Atg7 (E1-like) and Atg10 (E2-like). The Atg5-Atg12 complex then binds Atg16L1, resulting in an Atg12-Atg5-Atg16L1 complex that attaches to the autophagosomal membranes. Second, LC3-I is generated from the LC3 precursor mediated by proteolytic cleavage by Atg4 and becomes lipidated in a series of ubiquitin-like reactions executed by E1-like (Atg7), E2-like (Atg3) and E3-like (Atg12-Atg5-Atg16L1) enzymes, resulting in LC3-II [11]. These reactions are crucial for the autophagy process, as Atg5-, Atg7-deficient mice and cells lack autophagic features [23, 24], although in specific tissues, Atg5 and Atg7-independent autophagy has been described [25]. LC3-II attaches to the autophagosomal membrane. This is exploited to determine the amount of autophagosomes by LC3-II detection. However, this does not necessarily reflect autophagic activity, since an inhibition of autophagosome-lysosome fusion too can lead to an accumulation (and thus increase) of autophagosomes [26]. As such, LC3 data are often obtained in the absence and presence of lysosomal inhibitors and combined with experiments determining the levels of Sqstm1/p62, a long-lived protein, in order to carefully assess autophagic flux. This autophagy receptor links the cargo (e.g. polyubiquitin) to LC3-II at the autophagosomal membranes to be sequestered. Ultimately, p62 is degraded in the lysosomes, making its levels a valuable marker for the autophagic degradation rate [27].

Here, we will focus on the emerging role of autophagy as a novel connexin-turnover pathway with a brief summary of the earlier work implicating proteasomal and endosomal/lysosomal pathways for which we would like to refer to the many excellent and recent reviews on this topic published elsewhere [28–31].

## Connexin turnover and degradation by proteasomal and lysosomal pathways

Connexin proteins, together with pannexins and CALHM1, form the basis for intercellular communication between cells and cell types in different tissues and organs [32, 33]. Hexameric connexin structures, called “connexons”, form the functional units, being unopposed hemichannels or head-to-head docked gap junctions, providing flux pathways for a variety of ions, amino acids, nucleotides, metabolites and signaling molecules with molecular weights below 1.5 kDa and small RNA molecules. Cx43, which represents the 43-kDa connexin protein, is the most abundant connexin ubiquitously expressed in a plethora of tissues and organs. As gap junctions, connexins control a plethora of cellular and physiological processes, including the regulation of cell growth, migration, apoptosis, and cell signaling [34–37]. Gap junction dysregulation has been implicated in different pathophysiological conditions, including ischemia/reperfusion in the heart, epilepsy, inflammation, neurodegeneration and myocardial infarctions [38–40]. Different mutations in connexin-encoding genes causing dysfunctional, mislocalized or downregulated gap junctions have been linked to human diseases such as cardiovascular diseases, congenital deafness, skin diseases, myelin-related diseases and cataracts [41–43]. Recently, hemichannels too have been emerging as important signaling hubs not only in pathophysiology (like ischemia reperfusion in brain and heart tissues), but also in physiology [44], like in fear memory consolidation in astroglia of the basolateral amygdala [45], in osteocyte viability important for bone structure, integrity and function [46, 47] and as natural oncosuppressors that counteract cancer growth and metastasis to the bone [48].

Importantly, connexin gap junction complexes are very dynamically different regulated in a variety of physiological and pathophysiological conditions in different cell types and systems (reviewed in [49]). In comparison to other membrane proteins characterized by half-lives between 17 and 100 h, the turnover of connexin proteins is very rapid, between 1.5 and 5 h in various cell lines, cell types and tissue [49]. Rapid degradation may be an important mechanism for adjusting intercellular coupling of cells under normal and pathophysiological conditions. As an exception, a subpopulation of connexins expressed in the lens, like Cx45.6, Cx46 and Cx56 has a prolonged half-life (up to days) [49]. The turnover of connexin gap junctions is also dynamically regulated in response to physiological and pathophysiological conditions, including the response to EGF, phorbolesters or injury. This involves a well-characterized cascade of phosphorylation events [50]. For instance, Akt phosphorylation on Cx43 Ser373 promotes Cx43 gap junction assembly in an open state in response to EGF/injury, which is followed by a MAPK-mediated phosphorylation of Ser278/Ser282, closing gap junction, and a Src-mediated phosphorylation of Tyr247 causing Cx43 gap junction internalization [50, 51]. Degradation of Cx43 gap junctions may occur via the degradation of the “oldest subdomains” (i.e. containing the “oldest proteins”) within the gap junction or via the internalization of the complete gap junction in a double membranous structure, called an “annular” junction or “connexisome” [50, 51]. This internalization of Cx43 gap junctions occurs at least in part via small endocytic double-membrane vesicles [52] in a clathrin-dependent manner [53, 54]. A plethora of studies revealed that the degradation of Cx43 proteins involve proteasomal and lysosomal pathways, including autophagy. The dynamic regulation of Cx43 gap junction size and levels by phosphorylation events triggered by physiological and pathophysiological stimuli is discussed elsewhere in different excellent reviews by others [50, 51].

Seminal work by Beyer and his team showed a prominent role for the ubiquitin-dependent proteasome as a major connexin-turnover pathway in normal conditions and stress conditions like heat stress [55]. Inhibitors of the proteasome significantly increased the half-life of Cx43 proteins in pulse-chase experiments. Besides pharmacological approaches, his team also used genetic-based approaches thereby elegantly comparing the turnover of Cx43 in E36 chinese hamster ovary cells and in E36 cells containing a temperature-sensitive version of the ubiquitin-activating protein, E1 (ts-E36). The ts-E36 cells are defective in the proteasomal degradation of short-lived or abnormal proteins at high temperatures (39 °C) but not at low temperatures (30 °C). While Cx43-protein levels were similar in E36 and ts-E36 cells at low temperatures, Cx43-protein levels were prominently higher in ts-E36 cells than in wild-type E36 cells at high temperatures. The decline of Cx43 in E36 cells could be rescued by proteasomal inhibitors. Consistent with these findings, Cx43-ubiquitin conjugates could be identified in lysates of E36 cells treated with proteasomal inhibitors. A similar role for proteasomal degradation of Cx43 was described in rat heart-derived BWEM cells in which the loss of plasmalemmal Cx43 triggered by treating the cells with brefeldin A or monensin could be prevented by proteasomal inhibitors. Follow-up studies based on primary cultures from neonatal rat ventricular myocytes demonstrated that heat stress could enhance Cx43 degradation by the proteasome [56]. Interestingly, heat shock protein 70, upregulated in response to heat stress, emerged as a protective factor preventing Cx43 degradation upon repeated heat stress stimuli.

**Fig. 2:**
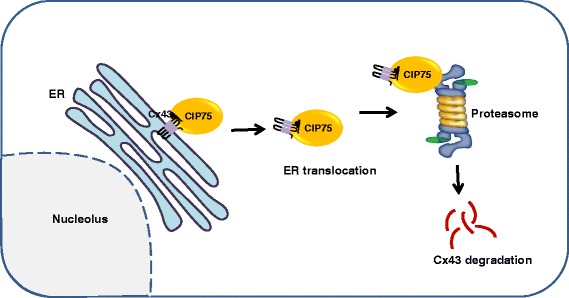
Proteasomal degradation of Cx43 via ERAD: CIP75 binds to the C-terminal tail of ER-localized Cx43 via its UBA domain, and then facilitates the translocation of Cx43 from the ER membrane into the cytosol. This complex interacts with 19S proteasome subunit via the CIP75 UBL domain, leads to the proteasomal degradation

The proteasome not only appears to be important for plasmalemmal Cx43 degradation, but likely assists in the ER-associated degradation (ERAD) mechanism (Fig. 2) of Cx43 dislocated from the ER of cells exposed to cellular stress (like in cells exposed to the reducing agent DTT) [28, 57]. Further molecular studies provided evidence that ER-localized Cx43 could directly interact with a 75-kDa protein [58, 59], named Cx43-interacting protein of 75-kDa or CIP75. This protein belongs to the ubiquitin-like (UbL) and ubiquitin-associated (UBA) domain protein family [60]. UBL/UBA-domain-containing proteins serve as shuttle/adapter proteins interacting on the one hand with ubiquitinated targets and on the other hand with the Rpn1 and Rpn10 subunits of the 19S regulatory particle, which together with the 20S core particle forms the 26S proteasome holoenzyme [58, 60]. Here, the UBA domain of CIP75 directly bound to the carboxyl terminus (CT) of Cx43 via a low-affinity interaction (K_d_ of about 300 μM), involving several regions within the intrinsically disorder part of the CT tail of Cx43 [61]. Further studies confirmed the presence of a multi-protein complex involving ER-localized Cx43/CIP75/Rpn1/Rpn10 [62] in which CIP75 is essential for the interaction of Cx43 with the proteasomal components. CIP75 controlled Cx43 half-life, which increased in the absence of CIP75 and decreased upon CIP75 overexpression [58]. CIP75-induced Cx43 degradation could be prevented by proteasomal inhibitors. Interestingly, although CIP75 directly interacts with different ubiquitinated proteins, including the well-documented p27 (Cip/Kip family of cyclin dependent kinase inhibitor 1B (CDKN1B)), to direct them to the proteasome for degradation, the interaction of CIP75 with ER-localized Cx43 occurred in an ubiquitin-independent manner [59]. Indeed, ER-localized Cx43 co-immunoprecipitated with CIP75 was not ubiquitinated, while immunoprecipitation of ubiquitinated proteins did not yield any Cx43. This finding was corroborated by experiments showing the binding of CIP75 to lysine-mutated Cx43, void of potential ubiquitin-acceptor sites. Further experiments indicated that the interaction of CIP75 with Cx43 was increased upon misfolding of the Cx43 protein, either induced by mutations or by DTT [62]. Hence, CIP75 was proposed to facilitate the dislocation of Cx43 from the ER membranes into the cytosol, where it can be targeted to the proteasome for degradation by associating with the Rpn1 and Rpn10 components [62]. Very recently, the role of CIP75 in the proteasomal degradation of connexin proteins appeared not to be limited to the Cx43 isoform, but also impacted the degradation of other connexin isoforms, including Cx40 and Cx45 [61]. Similarly as for Cx43, the UBA domain of CIP75 was found to directly interact with the CT regions of Cx40 and Cx45 via low-affinity interactions involving several intrinsically disordered regions, which were not conserved among the different Cx43, Cx40 and Cx45 proteins. Consistent with these molecular findings, CIP75 levels affected the degradation of Cx40 and Cx45 proteins, in which CIP75 shRNAs augmented Cx40- and Cx45-protein levels, likely by hampering their proteasomal degradation via ERAD. This is supported by the fact that cells expressing ER-targeted Cx40 or Cx45, treated with brefeldin A/DTT to induce Cx40 or Cx45 misfolding and degradation by ERAD, displayed augmented Cx40/CIP75 or Cx45/CIP75-complex formation and co-localization at the ER membranes. Besides Cx40, Cx43 and Cx45, also Cx32 appears to be degraded via the proteasome involving ERAD, but this likely involves the canonical ubiquitination pathway of Cx32 [57, 63, 64]. This appears to be supported by biochemical evidence that CIP75 could be co-immunoprecipitated with cellular Cx32 but not with the bacterially expressed and purified CT of Cx32 [61]. Proteasomal degradation has also been implicated in the turnover of pathological mutant connexin channels that are inherently unstable [65]. For instance, a frame shift mutation in Cx50 causing recessive congenital cataract resulted in an unstable Cx50 protein that is rapidly degraded via ERAD and the proteasome, thereby reducing Cx50 gap junctions and impairing intercellular communication [65]. Epoxomicin, a proteasomal inhibitor, prevented the degradation of the mutant Cx50 protein, increasing Cx50-protein levels and their presence as functional gap junctions, capable of restoring intercellular coupling.

**Fig. 3:**
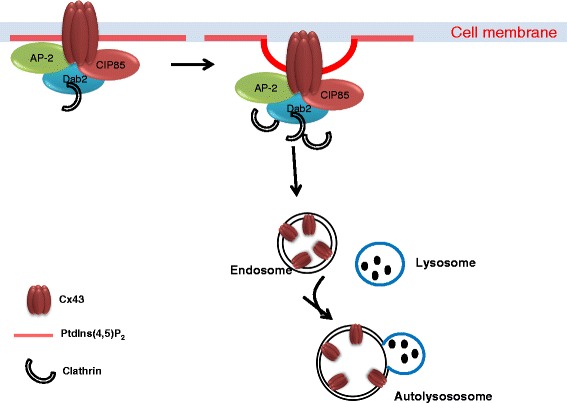
Endolysosomal degradation: CIP85 interacts with Cx43 via SH3 domain at plasma membrane. Initiation of clathrin-mediated endocytosis starts at PtdIns(4,5)P_2_-rich sites in the plasma membrane where the AP2 adaptor complex associates and recruits further accessory proteins. AP2 complexes connect the cargo membrane protein to clathrin coat. GTPase dynamin is necessary for movement of the vesicle and cargo into the cell. Internalized Cx43 are degraded via lysosomal degradation

However, early on in connexin research, it became also very clear that lysosomal pathways contributed to the connexin degradation, potentially as a minor component in normal physiological conditions, but gaining more importance upon stress [55, 66]. Lysosomotropic amines slightly increased the half-life of Cx43 in response to heat stress [55]. However, follow-up studies showed that endogenous Cx43 levels were about 4-fold higher in BWEM cells exposed to proteosomal or lysosomal inhibitors than in untreated BWEM cells [66]. In brefeldin A-induced Cx43-degradation experiments, proteasomal and lysosomal inhibitors appeared both effective in counteracting Cx43 degradation. Also, brefeldin A caused internalization of Cx43 gap junction, as a step preceding degradation. Strikingly, proteasomal inhibitors could largely restore the presence of Cx43 in gap junctional plaques at appositional membranes in brefeldin A-treated cells, while lysosomal inhibitors were much less effective. In the latter, Cx43 gap junctions mainly appeared in intracellular vesicles. Different studies provided evidence for endolysosomal Cx43 degradation (reviewed in [28]). Plasmalemmal Cx43 gap junctions internalized via the endocytic pathway and subsequently fused with lysosomal structures (Fig. 3). As discussed elsewhere [29], Cx43 internalization depended on the presence of clathrin, clathrin-adaptor proteins (including AP-2 and Dab2) and dynamin 2 and on dynamin GTPase activity. Also, the Cx43-interacting protein of 85 kDa, CIP85, an ubiquitously expressed Rab GTPase-activating protein, emerged as a critical factor for Cx43 internalization and lysosomal degradation [67]. CIP85 co-localized with Cx43 at the cell surface by means of a direct interaction between CIP85 and Cx43, involving the SH3 domain and the proline-rich region P^253^LSP^256^, respectively. Overexpression of CIP85, but not CIP85 lacking its SH3 domain, increased Cx43 turnover likely by recruiting clathrin and subsequently enhancing Cx43 internalization as gap junctions from the plasma membrane [67]. Strikingly, lysosomal inhibitors, such as NH_4_Cl or leupeptin, but not proteasomal inhibitors, such as MG132, prevented the increased turnover of Cx43 by CIP85 overexpression [68]. Further studies showed the accumulation of connexins in lysosomes in cells exposed to lysosomal inhibitors, preventing their degradation and correlating with an increased appearance of Cx43 at the cell surface in such conditions [69].

## Autophagy: an emerging connexin-degradation pathway

Besides the endosomal/lysosomal pathway, in recent years, autophagy emerged as an important connexin-degradation pathway. This is supported by electron microscopic observations made about 30 years ago showing the presence of gap junctions in autophagic structures, the typical double membrane vesicles containing cellular material [52]. However, only recently, mechanistic insights in the role of autophagy for connexin turnover have been obtained.

### Basic molecular mechanisms underlying connexin degradation by autophagy

The first direct evidence for a role of autophagy in connexin degradation was provided by the work of Beyer and his team [70]. Nutrient starvation, a potent inducer of the canonical mTOR-controlled autophagy pathway, triggered a rapid decline in the protein levels of wild-type Cx50, ectopically expressed in HeLa cells, and of wild-type Cx43, endogenously expressed in NRK cells. Nutrient starvation periods of just 2 h already impacted Cx43- and Cx50-protein levels. This corresponded with a marked decline in Cx43 and Cx50 levels at the plasma membrane and in perinuclear regions and a prominent increase in cytosolic LC3-positive autophagosomal punctae that stained positive for Cx50. About 1/3 of the intracellular Cx50 co-localized with LC3 in cells undergoing starvation for 4 h. Also, p62, another autophagic marker, could be detected in punctae that co-localized with intracellular, but not plasmalemmal, Cx50 or Cx43. Importantly, intracellular connexin gap junctions appeared in intracellular double membrane vesicles that were in close proximity of the endoplasmic reticulum, a hall-mark of autophagosome formation and autophagy. Basal autophagy appeared to contribute to the normal turn-over of Cx50 and Cx43 proteins, since chloroquine, a lysosomal inhibitor, caused an increase of about 30 % in basal Cx50-protein levels in transfected HeLa cells and of more than 100 % in basal, endogenous Cx43-protein levels in NRK cells, both grown in normal, nutrient-rich medium conditions. In these cell systems, chloroquine largely prevented the starvation-induced decline in Cx50- and Cx43-protein levels. These observations correlated with the co-localization of LC3 with both Cx50 and Cx43 under normal growth conditions, suggesting connexin turnover by basal autophagy and an increased co-localization during starvation periods (in the presence of chloroquine to prevent connexin degradation). Also depleting cells of Atg (autophagy-related) proteins, essential for autophagic flux, impacted connexin-protein levels. Knocking down Atg5 caused an increase of about 50 % in the basal Cx50-protein levels in non-starved HeLa cells and largely prevented the starvation-induced decline in Cx50-protein levels in starved HeLa cells. Similar results were obtained for Cx43 in MEF cells, in which Atg5-knockout MEF did not display any decline in Cx43-protein levels upon starvation. This study also implicated autophagy as an important degradation pathway for mutant Cx50 proteins, like Cx50^P88S^ associated with cataract. In fluorescent microscopy experiments, cytoplasmic accumulation of Cx50^P88S^ co-localized with the endogenous autophagosomal markers LC3 and p62, and with the lysosomal markers LAMP1. Consistently, starvation caused a major decline in the Cx50P88S-protein levels compared to non-starved conditions. The co-localization of p62 with Cx43 and Cx50 might imply a role for p62 in targeting connexins to the autophagic machinery for degradation, given p62’s role as a linker protein between ubiquitinated proteins and LC3.

**Fig. 4:**
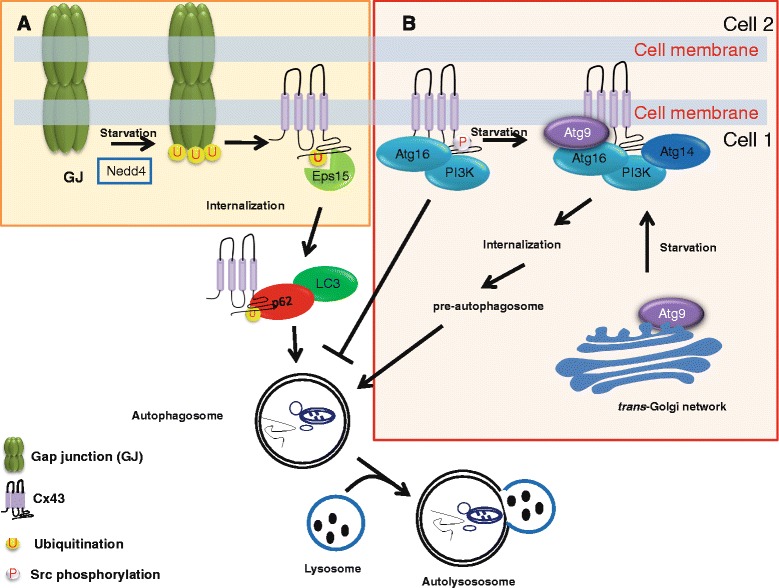
Connexin degradation by autophagy pathway: **a** Nedd4 mediates ubiquitination of Cx43 (shown Cx43 subunit) in gap junction plaques and recruits Eps15 to gap junctions upon nutrient starvation. Eps15 can interact with Cx43 via ubiquitin-binding domain, thereby promoting its internalization. Upon starvation, Cx43-p62-LC3 interaction can direct Cx43 for autophagy degradation. **b** In normal conditions, autophagosome precursor proteins such as PI3K complex (Beclin 1, Vps34 and Vps15) and Atg16 are associated with Cx43 (as Cx-Atg complex) at the plasma membrane. The Cx-Atg complex negatively regulates autophagy. During starvation, the arrival of Atg14 and Atg9 to the Cx43-enriched plasma membrane, activates Vps34 kinase, thereby inducing internalization of the Cx-Atg complex and the delivery of these pre-autophagosomal components to the sites of autophagosome formation. Internalization of Cx43 requires the presence of Eps15 (not shown). For clarity reasons, the internalization of only one Cx43 subunit is shown in both channels

In subsequent work by Cuervo and co-workers [71], molecular determinants controlling Cx43 degradation by autophagy were elucidated. In this study, it was shown that several endogenously expressed connexins, Cx26, Cx32 and Cx43, in the liver were present in autophagic vesicles (autophagosomes and autophagolysosomes) and co-fractionated with LC3. Connexin accumulation in the autophagic vesicles became more pronounced in liver samples starved for 6 h and in cultured cells from which serum was removed. Depletion of essential autophagy proteins in cells by Atg7 knockdown or Atg5 knockout prevented the degradation of connexins in response to serum removal. Similar effects were obtained using 3-methyladenine, a PI3K inhibitor that inhibits autophagy by suppressing autophagosome formation. Using photoswitchable Dendra-fused connexins to track *de novo* synthesized proteins as red fluorescent proteins, it was observed that serum-starvation caused the rapid degradation of Cx26, Cx32 and Cx43. However, in contrast to Cx26 and Cx32, Cx43 could be completely rescued by Atg7 knockdown, implying different roles for autophagy in the degradation of different connexin isoforms. Consistent with these findings, serum removal caused Cx43 relocalization from the plasma membrane to autophagosomes. The presence of Cx43 in these LC3/p62-positive vesicles was enhanced upon treatment with lysosomal inhibitors. In serum removal conditions, the degradation of Cx43 appeared to be mediated via the lysosomes but not the proteasome, since proteasomal inhibitors (MG132 or lactacystin) did not prevent the decline in Cx43-protein levels. Further cell surface biotinylation and confocal microscopy experiments revealed that mainly Cx43 gap junctions are rapidly degraded by autophagy in conditions of nutrient starvation. As a consequence, Cx43 remained present in the plasma membrane of starved cells treated with 3-MA or in which Atg5 or Atg7 was ablated. These findings were supported by in vivo evidence in liver-specific ATG7-knockout mice, displaying increased Cx43 levels and enhanced appearance of Cx43 at the plasma membrane. These molecular findings were also supported by evidence obtained at the functional level. Intercellular dye spreading, a measure for gap junctional coupling, was markedly declined upon nutrient starvation, while this decline could be alleviated upon chemical or genetic inhibition of autophagy. In addition, a link between internalization and autophagy was established. Indeed, lindane caused Cx43 internalization and degradation involving a time-dependent and autophagy-dependent redistribution of Cx43 from the plasma membrane to late endosomal/lysosomal vesicles identified by LAMP-1. The requirement of Cx43 internalization for its autophagic degradation was further elegantly illustrated by the use of the endocytosis-deficient Cx43^Y286A^ mutant. Interestingly, in contrast to wild-type GFP-Cx43, GFP-Cx43^Y286A^ persisted at gap junctions in the plasma membrane in starved cells. The internalization and autophagic degradation of Cx43 appeared to be dependent on ubiquitination of Cx43 by Nedd4 (Fig. 4a) and the further recruitment of the endocytic protein epidermal growth factor receptor substrate 15 (Eps15). Nedd4, an ubiquitin ligase enzyme, was previously implicated in binding the C-terminus of Cx43 and mediating Cx43 ubiquitination [72–74]. Eps15, an endocytic adaptor protein containing ubiquitin-binding domain, was previously identified to interact with ubiquitinated Cx43 and promote its internalization [74]. Serum starvation induced Nedd4-dependent ubiquitination of Cx43, but not of the Cx43^Y286A^ mutant, before its accumulation in autophagosomes [71]. Cx43 expressed in Nedd4-deficient cells was resistant to degradation by nutrient starvation. These findings were corroborated by showing that ectopically expressed Cx43, but not Cx43^Y286A^, fused to a single ubiquitin molecule was rapidly degraded in an autophagy-dependent manner in response to nutrient starvation. It was here confirmed that p62 served as cargo-recognition factor, forming a bridge between ubiquitinated Cx43 and LC3. Cx43-p62 interaction was enhanced during starvation, but was strongly diminished in cells lacking Nedd4. Furthermore, a critical role for Eps15 was identified in the early steps of Cx43 degradation.Cx43-Eps15 interaction was enhanced upon starvation, directing Cx43 for autophagic degradation. In cells lacking Eps15, Cx43 persisted at gap junctions in the plasma membrane upon nutrient starvation. Cx43-Eps15 interaction did not require autophagy, since cells lacking Atg5 or Atg7 or treated with 3-MA even displayed higher levels of Cx43-Eps15-complex formation. However, ubiquitination of Cx43 was critical for Eps15 interaction, since Cx43^Y286A^ and Cx43 expressed in Nedd4-deficient cells failed to recruit Eps15 in response to serum starvation. Excitingly, in this study [71], Eps15 was also identified as a novel autophagy cargo recognition molecule, given its ability to interact with LC3 in LC3-immunoprecipitation assays and its ability to recruit LC3 and Cx43 in Eps15-immunoprecipitation assays. In any case, these data revealed autophagy as a major turn-over pathway for Cx43 via a mechanism that involves Nedd4-dependent ubiquitination, recruitment of Eps15 and associations with the autophagic machinery, thereby facilitating Cx43 gap junction internalization and autophagic degradation via the lysosomes.

A detailed ultrastructural, microscopy-based study also revealed autophagy as a major clearance/degradation pathway for internalized annular gap junctions from the cytoplasm [75]. Internalized annular gap junctions appeared to co-localize with autophagosomal cargo, sequestration and degradation markers, like p62, RAB-7 and LC3. Double-membrane phagophore formation could be observed around annular gap junctions, eventually resulting in lysosomal degradation. Interfering with autophagy using pharmacological agents or by knocking down essential autophagy or cargo proteins increased total Cx43-protein levels and augmented the number of Cx43-containing annular gap junctions. It was postulated that these annular gap junctions resemble large and complex protein/vesicular structures (e.g. like protein aggregates, multi-protein complexes, damaged organelles and pathogens) that are typically removed from the cytoplasm via autophagy rather than via the proteasome [75]. However, a subset of the annular gap junctions could be directed to the lysosomes via the endosomal system, as has been observed for hyperphosphorylated and hyperubiquitinated Cx43 in response to chemical PKC hyperactivation [76]. Shuttling of ubiquitinated Cx43 from endosomes to lysosomes appears to be dependent on two ubiquitin-binding proteins, namely Hrs (hepatocyte growth factor-regulated tyrosine kinase substrate; also known as Hgs) and Tsg101 (tumor susceptibility gene 101) [77]. Another recent ultrastructural study revealed that annular gap junctions could be recycled to the plasma membrane or degraded via three pathways, including endo-lysososomal pathways, autophagosomal pathways and a direct engulfment by lysosomes [78].

### Connexin degradation by autophagy in different cellular and physiological systems and pathophysiological conditions

An increasing number of studies report an emerging role for autophagy as the turnover pathway of connexins in pathophysiological conditions and diseases, including ischemic insults and cancers.

#### Low pH in pancreatic acinar cells

Besides starvation, low pH, a condition known to uncouple connexin gap junctions in heart [79], has been implicated in the degradation of Cx32 gap junctions present in pancreatic acinar cells [80]. In particular, low pH in combination with cerulein, which triggers IP_3_-induced Ca^2+^ oscillations synchronized by gap junctions, caused a striking decline in Cx32-protein levels and Cx32 gap junction-mediated coupling of acinar cells. In these conditions, Cx32 gap junctions were lost and rapidly turned over, correlating with a punctate intracellular localization. The decline in Cx32-protein levels by the low pH/cerulean treatment could be prevented by lysosomal inhibitors and by the PI3K/autophagy inhibitor 3-MA. However, also proteasomal degradation pathways appeared to contribute to Cx32 degradation in this system. In another study, cadmium-induced toxicity in BRL 3A rat liver cells was linked to an increase in autophagic markers and an upregulation of essential autophagy proteins Atg5, Atg7 and Beclin 1. This coincided with a decrease in Cx43-protein levels and gap junctional coupling, which could be counteracted by lysosomal inhibitors and aggravated by the autophagy-inducer rapamycin.

#### Heart failure and ischemia in the heart

A first study focusing on normal and failing heart was able to link autophagic markers to lateralized and subsequently internalized Cx43 gap junctions in cardiomyocytes [81]. In normal hearts, Cx43 gap junctions appear at the intercalated disc region of the cardiomyocyte plasma membrane to properly propagate and spread electrical signals coordinating heart contractions. In failing hearts, Cx43 gap junctions relocalize to lateral cell membranes. In these failing hearts, Cx43 gap junctions became internalized and appeared in multilamellar membrane structures. The membranes appeared to contain buoyant, cholesterol and sphingolipid-rich membrane domains (referred to as lipid raft or LR membrane domains), in which a subset of the Cx43 gap junction population can be accumulating. Interestingly, in failing hearts, the total Cx43 protein levels declined correlating with an increased accumulation of the Cx43 protein in these LR membrane domains compared to the Cx43 levels in LR membrane domains from normal hearts. These structures also resembled the properties of autophagosomes and accumulated the lipidated protein LC3-II, which too became upregulated in failing hearts. As such, the LR fractions contained both Cx43 and LC3 protein. Interestingly, Cx43 and GFP-LC3 co-localized in intracellular punctae resembling autophagosomes in HeLa cells and in neonatal rat ventricular cardiomyocytes. However, this study did not show whether autophagy or its increased flux was responsible for the turnover and degradation of the Cx43 gap junctions.

Further work on the cardiac system focused on the role of autophagy in degrading Cx43 upon ischemia and ischemia/reperfusion. Ischemia/reperfusion has been associated with excessive autophagy induction in different physiological systems like the heart [82], liver [83] and kidneys [84], resulting in detrimental effects like autophagy-dependent cell death or autosis [85]. In the cardiomyocyte cell line HL-1, ischemia diminished total Cx43-protein levels and plasmalemmal Cx43 levels, including hemichannels and gap junctions [86]. This decline in Cx43 could be partially rescued by lysosomal inhibitor Bafilomycin A, implying ischemia-induced Cx43 degradation by autophagy in HL-1 cells. These observations correlated with an increased appearance of autophagosomes, LC3-II accumulation, GFP-LC3-punctae formation and Cx43-LC3 co-localization in HL-1 during ischemia. These findings were also observed in organotypic heart slices and in Langendorff heart preparations. Functionally, ischemia in HL-1 reduced intercellular coupling via gap junctions, which could be rescued by the autophagy inhibitor 3-MA. Consistent with previous studies, a “proximal” role for Eps15 was found for the internalization of Cx43 that became ubiquitinated during ischemia and a more “distal” role for p62 for the targeting of the internalized, ubiquitinated Cx43 to the autophagic machinery. Unexpectedly, while Nedd4 controlled ubiquitination of Cx43 and impacted basal Cx43 level in HL-1 cells, ischemia-induced ubiquitination and degradation of Cx43 was not altered in HL-1 cells in which Nedd4 was knocked down. This suggests that other E3 ubiquitin ligases may be involved in ubiquitination of Cx43 during ischemia. Also ischemia/reperfusion caused Cx43 degradation in a more excessive manner than ischemia alone. Cx43 degradation too relied on autophagy, although 3-MA added only during reperfusion could not prevent Cx43 degradation, implying an important role for Cx43-containing autophagosomes that have been accumulating during the ischemia period. Interestingly, AMP-activated kinase (AMPK) and Beclin 1 appeared to play different key roles in the Cx43 degradation upon ischemia versus ischemia reperfusion. Early on during ischemia, AMPK is robustly activated, implying a marked decline in cellular ATP levels. Chemical inhibition of AMPK could partially prevent ischemia-induced Cx43 degradation during these early periods of ischemia. Extended periods of ischemia led to a decline in AMPK activity and a failure of the AMPK inhibitor to stabilize Cx43. During these extended periods, Beclin 1 appeared important, since siRNA against Beclin 1 could prevent Cx43 degradation during the longer ischemia periods. During ischemia/reperfusion, only Beclin 1 siRNA but not AMPK inhibition was effective in preventing Cx43 degradation. Thus, these data imply different molecular autophagy key players become activated during the different periods of ischemia and ischemia/reperfusion, ultimately controlling Cx43 degradation. Very interestingly, a recent study aiming to document the proteomic changes associated with the susceptibility of neonatal versus adult murine hearts to ischemia/reperfusion reported an increase in Cx43 levels but a decrease in Beclin 1 levels upon maturation [87].

#### Oncogenesis in different tumors

Both altered autophagy [88, 89] and altered connexin expression [90–92] have been implicated in oncogenesis in a variety of tumors. In particular, in a variety of tumors connexin gap junctions appear to be downregulated [93], yet the contribution of autophagy in oncogenic connexin downregulation is definitely not clear. Nevertheless, a number of studies appear to suggest the involvement of autophagy in connexin turnover in tumors.

For instance, in non-small cell lung cancer cells (NSCLC), autophagy is responsible for the rapid degradation and downregulation of the Cx31.1. isoform. In H1299, a NSCLC cell line, Cx31.1 overexpression resulted in an intracellular localization of the protein, consistent with the very rapid turn-over of Cx31.1 in these cells. The rapid turnover of Cx31.1 could only be partially prevented by proteasomal inhibitors. Moreover, autophagy-inducing conditions like starvation and brefeldinA further increased the rapid decline in Cx31.1 that was only mildly impacted by proteasomal inhibitors. In contrast, knockdown of Atg5 prominently protected Cx31.1 against starvation-induced degradation. In these cells, Cx31.1 appeared in intracellular punctae that co-localized with the autophagic marker LC3 upon starvation. An unbiased mass spectrometry analysis of GFP-Cx31.1 immunoprecipitated samples revealed clathrin as a potential Cx31.1-binding partner, confirmed in direct co-immunoprecipitation assays. Cx31.1 and clathrin co-localized in cells, including in intracellular punctae that became more pronounced in starved cells. Knockdown of clathrin appeared to augment basal Cx31.1 levels and prevented the starvation-induced decrease of Cx31.1 in these cells. Hence, this study shows that autophagy may contribute to the degradation of different connexin isoforms in cancer cells, although the impact of restoring Cx31.1 expression through autophagy inhibition on the oncogenic properties of these tumors ought to be further characterized.

A further role for altered connexin presence in tumors linked to autophagy has been observed in keratocystic odontogenic tumors [94]. In these tumor samples, Cx43 and Cx32 levels were significantly downregulated compared to normal oral mucosa samples. The Cx43 and Cx32 levels negatively correlated with the expression of the autophagy markers LC3 and p62. This may imply that excessive autophagy and upregulation of autophagy markers linked to both Cx43 and Cx32 degradation could contribute to the downregulation of Cx43 and Cx32 in these tumors. Thus, inhibiting autophagy may have a tumor suppressive potential in these patients by restoring normal Cx43 and Cx32 levels.

Connexin degradation by autophagy also has been implicated as an escape mechanism of tumor cells for immune surveillance by natural killer cells [95]. In particular, hypoxic stress, occurring in the tumor micro-environment, appears to be involved in escaping immune surveillance, including a suppressed susceptibility of tumor cell lysis by natural killer cells [96]. While hypoxic stress increased the total Cx43-protein levels in a HIF1α-driven manner in melanoma cells, the presence of Cx43 gap junctions at the immunological synapse between melanoma cells and natural killer cells was strongly diminished [95]. The decline of Cx43 gap junctions at the immunological sequence was dependent on the increased autophagic flux occurring during hypoxia. Indeed, the presence of Cx43 at the immunological synapse could be restored by inhibiting lysosomes or knocking down Atg5. Importantly, inhibiting autophagy and thus preventing the subsequent degradation of Cx43 at the immunological synapse was very effective in restoring the susceptibility of hypoxic melanoma cells towards natural killer cell-mediated lysis. These findings were corroborated by the use of the endocytic Cx43^Y286A^ mutant, which remained present at the immunological synapse during hypoxic stress and also restored the susceptibility of tumor cells towards lysis by natural killer cells in hypoxic conditions. Thus, this study suggests that inhibition of autophagy may serve as a strategy to render tumors more vulnerable to attacks by the immune system (e.g. through lysis by natural killer cells) by restoring the presence of connexin at immunological synapses.

### Relevance of connexin degradation by autophagy

The cellular and physiological relevance of connexin degradation by autophagy, in particular in response to stress conditions like nutrient starvation, remains to be further explored. In general, it is proposed that autophagy activation as an initial response to stress serves as a cell survival pathway while evading cell death. However, excessive or prolonged autophagy will lead to and/or contribute to cell death. Also, autosis, a new form of cell death associated with high levels of autophagy, has been described [85]. Furthermore, there are several mechanistic links between autophagy and several cell death processes [85, 97]. For instance, anti-apoptotic Bcl-2 proteins can bind Beclin 1, thereby preventing its pro-autophagic function and thus co-regulating apoptosis and autophagy [98]. Also, receptor-interacting protein kinase-1 (RIPK1), a signaling node in the control of apoptosis and necroptosis in response to extrinsic death factors, suppresses basal autophagy by promoting ERK-dependent phosphorylation of TFEB, thereby suppressing its transcriptional activity that is involved in the expression of genes with functions in autophagy and lysosomal biogenesis [99, 100]. Moreover, several proteins with pro-death functions have been shown to be degraded by autophagy [85]. For instance, FDA-approved inhibitors of mTOR decrease RIPK1 and RIPK3 levels in human renal cell carcinoma cells by activating autophagy, which underlies their resistance to these mTOR inhibitors [101]. Inhibition of autophagy using the lysosomal inhibitor chloroquine leads to RIPK1/RIPK3 stabilization in mTOR inhibitor-treated cells, thereby promoting necroptotic cell death in these cells. Autophagy also impacts the phosphatase FAP1 (Fas-associated phosphatase 1), which counteracts Fas/CD95-induced apoptotic cell death [102]. Cells with increased autophagy flux display reduced levels of FAP1, thereby sensitizing these cells to apoptosis elicited by Fas/CD95 signaling. Finally, also activated caspase 8 has been reported to be sequestered into autophagosomes and degraded in the lysosomes, thereby rendering Bax-deficient Hct116 cells resistant to TRAIL-induced apoptosis [103]. Inhibiting autophagy restored TRAIL-induced caspase 8-dependent apoptotic responses in these cells.

Importantly, connexin gap junctions too have been linked to cell death processes and were shown to be responsible for the spreading of cell death factors between neighboring cells involving the diffusion of IP_3_ as a critical factor [104, 105]. Connexin hemichannels may mediate the loss of essential survival factors and release of ATP, glutamate and prostaglandins as factors that participate in cell-death spreading through paracrine signaling [106]. Also, Cx43 gap junctions and ATP release contribute in vivo to the spreading of radiation-induced damage from the irradiated region (e.g. containing tumor cells) to other neighboring unexposed regions (e.g. healthy tissues) [107]. In that sense, the increased turnover of connexin proteins as pro-death signaling complexes by autophagy could be a manner to prevent cell death or cell death spreading during these early adaptive responses. Further work using strategies that favor the stabilization of connexin proteins during autophagy induction treatments and subsequently assess the impact on cell viability, susceptibility to cell death stimuli and cell death spreading should provide further insights in this process. In addition, Cx43 is also present in intracellular organelles, including the mitochondria, and can also control cell survival. Yet, their susceptibility towards stress conditions and autophagy degradation has not yet been studied. For a detailed discussion of the role of connexins as gap junctions and hemichannels or their channel-independent functions in controlling cell death and survival, we wish to refer to different reviews dealing with these topics [34, 36, 106].

At the physiological level, it will be interesting to understand how autophagy impacts connexin functions as gap junctions and hemichannels and connexin-mediated signaling processes in native tissues like brain, including the astroglial network, heart and bone. In particular, the use of tissue-specific knockouts for autophagy-dependent genes will be instrumental to unravel the physiological role of autophagy-dependent regulation of connexins, connexin-mediated signaling and connexin-controlled processes in vivo. Also, autophagy has been shown to decrease during ageing, to be impaired in different pathologies including neurodegenerative diseases and to play an anti-inflammatory role [108, 109]. Now, connexins too have been implicated in these processes with severe alterations in function and expression in several disease conditions [43, 110, 111]. For instance, the excessive opening of astroglial hemichannels has been implicated in response to inflammatory cytokines and amyloid β, leading to neuronal cell death [112–114]. Hence, alterations in autophagy might impact the surface level of connexins in gap junctions and hemichannels in these cell types and thus the pathophysiological outcome.

## Connexins: an emerging negative regulator of autophagic flux

While autophagy impacts connexin levels, it is also clear that connexins by themselves can suppress the autophagy process by recruiting pre-autophagosomal Atg proteins and PI3K components (Fig. 4b), as recently reported in a seminal paper by Cuervo and co-workers [115]. Cx43 could co-localize and associate with Atg16, a marker for pre-autophagosomal structures. The co-localization of Cx43 with Atg16 was not altered in cells undergoing nutrient starvation, in cells exposed with 3-MA or in cells lacking Atg15. The Cx43-Atg16 complex appeared prior to autophagy activation, since it did not contain Atg5. Importantly, mouse osteoblasts obtained from wild-type versus Cx43-knockout mice displayed altered autophagy. In the absence of Cx43, autophagic flux was enhanced with an increase in the number of autophagic vesicles and of steady state LC3-II levels. The inhibition of autophagy by Cx43 was independent of its ability to form functional gap junctions, since chemical inhibition of Cx43 gap junctions did not increase autophagic flux, while GFP-Cx43, a fusion protein that is located at the plasma membrane but that fails to form functional gap junctions, was fully capable of inhibiting autophagy. Interestingly, a C-terminally truncated Cx43 protein at position 258 too could repress autophagy correlating with its ability to interact with Atg16, while Cx43 truncated at position 245 failed to repress autophagy and to interact with Atg16. The 245–258 region also contains a Src-phosphorylation site. Inhibiting Src activity robustly increased autophagy in Cx43-expressing but not in Cx43-knockout cells, indicating a role for Src-dependent phosphorylation of Cx43 in suppressing autophagy. Besides Atg16, Cx43 also recruited different components of the class III PI3K complex. Different members of this initiation complex, Vps34, Beclin 1 and Vps15, were found to interact with Cx43 in normal, starved or rapamycin-treated conditions and independent on the expression of Atg5 or Atg16. Also other connexin isoforms appeared to negatively impact autophagic flux, indicating that many connexin-family members might serve as general autophagy regulators. Knockdown of Cx26 or Cx32 in MEF cells resulted in increased autophagy, coinciding with their ability to bind and recruit Atg16. Agents that promoted acute internalization of Cx43 also increased autophagic flux in a Cx43-dependent manner. Indeed, tamoxifen and lindane increased autophagy in Cx43-expressing cells, but not in Cx43-knockout cells, which displayed already high autophagy in basal conditions. The internalization of Cx43 also resulted in a decrease in Cx43-Atg16-complex formation, while Atg16-positive pre-autophagic compartments became more prevalent. Internalization of Cx43 appeared to be dependent on the recruitment of Atg9 and Atg14 towards Cx43-enriched plasma membrane regions. Upon starvation, several Atg9 (multimembrane-spanning protein) vesicles are redistributed from *trans*-Golgi network and interact with phagophores during early steps of autophagosome formation [116]. Both Atg9 and Atg14 must be present in these regions in a spatiotemporal manner before Cx43 gap junctions can be internalized and degraded by autophagy. Interestingly, the presence of Atg9 and Atg14 at the plasma membrane and their interaction with Cx43 was prominently increased upon conditions of serum starvation, explaining the increased Cx43 internalization during nutrient starvation. It is interesting to note that other channels have been implicated as regulators of the autophagy process by scaffolding/recruiting essential autophagy proteins. For instance, the inositol 1,4,5-trisphosphate receptor (IP_3_R), an intracellular Ca^2+^-release channel located at the endoplasmic reticulum, has been shown to recruit Beclin 1, thereby limiting its availability for the PI3K initiation complex and thus suppressing autophagy [117–119]. However, other studies proposed a stimulatory role for IP_3_Rs in autophagy by recruiting Beclin 1, sensitizing the IP_3_R channel to its agonist IP_3_ and promoting intracellular Ca^2+^-signaling events that are essential to upregulate autophagic flux in response to nutrient starvation [120–123].

Further studies have implicated that connexins could regulate the autophagy pathway. In hippocampal tissues obtained from rats exposed to traumatic brain injuries, astrocytic phosphorylated Cx43 levels increased, coinciding with an increase in the autophagic marker LC3-II in the neurons [124]. Carbenoxolone, a Cx43 gap junction/hemichannel inhibitor, prevented the increase in phosphorylated Cx43 caused by traumatic brain injury and also suppressed the increase in LC3-II levels. These experiments therefore suggested a link between the appearance of phosphorylated Cx43 in astrocytes and autophagy induction in neurons as a potential mechanism for brain injuries. This was further underpinned by in vivo experiments, in which both carbenoxolone and 3-MA could alleviate the memory deficits induced by traumatic brain injuries in an assay testing for spatial memory [125]. This correlated with a partial reversal of the loss of long-term potentiation in rat hippocampal slices induced by traumatic brain injury. Thus, both increased phosphorylated Cx43 and autophagy appeared to negatively impact brain function in this setup. At the molecular level, traumatic brain injury reduced the expression of the glutamate transporter GLT-1 and increased the expression of the ionotropic purinergic receptor P2X7R. Collectively, suppressing Cx43 phosphorylation by carbenoxolone, inhibiting P2X7R using oxATP or activating the GLT-1 transporter were able to counteract traumatic brain injury-induced Beclin 1 upregulation. Furthermore, phosphorylated Cx43 and the upregulation of the P2X7R were also able to prevent the injury-induced decline in GLT-1 levels. Collectively, these data imply a cascade in which the accumulation of phosphorylated Cx43 in astrocytes during brain injury leads to an upregulation of P2X7R, augmenting ATP release and ATP-induced signaling processes that result in a decrease in GLT-1 and excessive activation of the autophagic pathway via a marked upregulation of Beclin 1. This “toxic” signaling cascade appears to negatively impact brain functions after traumatic injury, since interfering with either Cx43 or autophagy is able to partially restore neuronal functions. However, the molecular mechanisms by which elevated levels of phosphorylated Cx43 in astrocytes upregulate autophagy in neurons require further studies.

## Conclusions

There is increasing evidence that autophagy and connexins are interrelated. On the one hand, autophagy is emerging as an important connexin-degradation pathway not only under basal and normal growth conditions and well-established autophagy-inducing conditions like nutrient starvation but also in a variety of pathological conditions. Autophagic degradation of Cx43 critically depends on Cx43 internalization, thereby requiring ubiquitination (e.g. by Nedd4), ubiquitin-binding proteins (e.g. Eps15), interactions with autophagic cargo proteins (e.g. p62), autophagosome markers (e.g. LC3) and other autophagy proteins that act early on in the process (e.g. Atg9 and Atg14). Furthermore, the degradation by autophagy is not limited to Cx43 but seems to contribute to the rapid turnover of many connexin isoforms, in particular during conditions of autophagy activation like nutrient starvation. In addition, autophagy is also important for the rapid turnover of mutant connexins associated with severe human diseases and altered autophagy in pathophysiological conditions can lead to impaired or enhanced connexin degradation. On the other hand, connexins are also emerging as novel negative regulators of the autophagy process by recruiting essential autophagy proteins to the plasma membrane, including Atg16 and PI3K-complex components like Vps34, Beclin 1 and Vps15 that act in the earlier steps of the autophagy process. As such, connexins can be limiting their availability for inducing autophagy upstream of autophagosome formation. As highlighted by Cuervo and co-workers [71, 115], the important interplay between connexins and autophagy is underscored by the fact that many connexin-associated diseases have been linked with altered autophagy, including cardiovascular diseases, ocular diseases, diabetes and cancer.
